# Quantitative SARS-CoV-2 Spike Receptor-Binding Domain and Neutralizing Antibody Titers in Previously Infected Persons, United States, January 2021–February 2022

**DOI:** 10.3201/eid3011.240043

**Published:** 2024-11

**Authors:** Anna Bratcher, Szu-Yu Kao, Kelly Chun, Christos J. Petropoulos, Adi V. Gundlapalli, Jefferson Jones, Kristie E.N. Clarke

**Affiliations:** Epidemic Intelligence Service, Centers for Disease Control and Prevention, Atlanta, Georgia, USA (A. Bratcher); Alan Shawn Feinstein College of Education, University of Rhode Island, Kingston, Rhode Island, USA (S.-Y. Kao); Labcorp Esoterix, Calabasas, California, USA (K. Chun); Labcorp-Monogram Biosciences, South San Francisco, California, USA (C.J. Petropoulos); Centers for Disease Control and Prevention, Atlanta (A.V. Gundlapalli, J. Jones, K.E.N. Clarke)

**Keywords:** COVID-19, respiratory infections, severe acute respiratory syndrome coronavirus 2, SARS-CoV-2, SARS, coronavirus disease, zoonoses, viruses, coronavirus, serosurveillance, binding antibodies, neutralizing antibodies, United States

## Abstract

We studied SARS-CoV-2 binding and neutralizing antibody titers among previously infected persons in the United States over time. We assayed SARS-CoV-2 spike protein receptor-binding domain and neutralizing antibody titers for a convenience sample of residual clinical serum specimens that had evidence of prior SARS-CoV-2 infection gathered during January 2021–February 2022. We correlated titers and examined them by age group (<18, 18–49, 50–64, and >65 years) across 4 different SARS-CoV-2 variant epochs. Among selected specimens, 30,967 had binding antibody titers and 744 had neutralizing titers available. Titers in specimens from children and adults correlated. In addition, mean binding antibody titers increased over time for all age groups, and mean neutralization titers increased over time for persons 16–49 and >65 years of age. Incorporating binding and neutralization antibody titers into infectious disease surveillance could provide a clearer picture of overall immunity and help target vaccination campaigns.

Since its emergence in late 2019, SARS-CoV-2 has posed a substantial challenge to public health surveillance worldwide. Seroprevalence, the proportion of a population that has detectable SARS-CoV-2 antibodies, has commonly been used to estimate population-level infection and vaccination history across geographic regions (*1*–*4*). However, seroprevalence studies only describe the presence or absence of antibodies. In addition, those studies generally refer only to binding antibodies, or antibodies that can recognize and attach to an antigen. However, binding antibody analyses neglect to describe the ability of antibodies to neutralize a pathogen, which is measured by neutralizing antibody titers. Therefore, additional information is needed when studying populations in which nearly all persons have detectible antibodies.

Binding and neutralizing antibody titers can be heterogeneous after SARS-CoV-2 infection or vaccination (*5*–*7*). Thus, those titers can be particularly valuable measures of protective immunity among populations where nearly all persons have detectible antibodies (*8*). In addition, the ability of those titers to detect heterogeneity in serologic status within fully seropositive populations, including changes in antibody levels over time, enables epidemiologists to provide a more nuanced description of serostatus for those populations. Furthermore, those antibody titers can clarify a population’s continued risk because evidence shows that higher binding and neutralizing antibody titers are associated with lower probability of infection, re-infection, and severe disease (*9*–*11*; J.A. Cohen et al., unpub. data, ). Moreover, understanding the correlation between the 2 measures can inform clinical practice. Strong evidence that neutralizing antibody titers are a correlate of protection is available, but acceptance of binding antibodies as a correlate of protection is less prevalent (*12*). Limited evidence is available on the correlation of binding and neutralizing antibody levels, particularly among a large, nationwide population spanning all age groups.

Measuring binding and neutralizing antibody titers after SARS-CoV-2 infection and vaccination also can enable the study of differences across key demographics. Using those measures, epidemiologists can statistically test antibody titers across characteristics such as age and sex that are likely to be associated with differences in immune response and severity of SARS-CoV-2 outcomes (*13*). In addition to enabling the study of risk factors, titers can help examine the immunogenicity of vaccination, booster vaccines, and reinfection (*14*,*15*), as well as the effects of waning antibodies over time (*16*). Measuring binding and neutralizing antibody titers can improve understanding of the course of the SARS-CoV-2 pandemic. As correlates of protection, those measures can be indicative of future transmission levels and risks for severe disease. To supplement understanding of seroprevalence over the COVID-19 pandemic, we studied binding and neutralizing antibody titers among previously infected persons in the United States during January 2021–February 2022.

## Methods

### Study Design and Sample

A convenience sample of residual clinical serum specimens were collected as part of the Nationwide Commercial Laboratory Seroprevalence Survey (NCLS; https://data.cdc.gov/Laboratory-Surveillance/Nationwide-Commercial-Laboratory-Seroprevalence-Su/d2tw-32xv/about_data). NCLS is a repeated, cross-sectional survey conducted by the Centers for Disease Control and Prevention (CDC) and designed to monitor national seroprevalence of infection-induced antibodies to SARS-CoV-2. Detailed methods are discussed elsewhere (*17*). In brief, we collected data from specimens submitted for clinical testing across 50 US states, the District of Columbia, and Puerto Rico. To minimize selection bias, we excluded specimens for which SARS-CoV-2 antibody testing was ordered by the clinician. Specimen information included data on patient age, patient sex, jurisdiction, and date of blood collection.

For this analysis, we included specimens gathered during January 2021–February 2022 that had antibodies against the nucleocapsid protein (anti-N). Anti-N are produced in response to infection but are not produced in response to the COVID-19 vaccines authorized for use in the United States at the time of data collection. Anti-N were detected by using the Elecsys Anti-SARS-CoV-2 assay (Roche Diagnostics, https://diagnostics.roche.com), which has a sensitivity of 100% and specificity of 99.8% for the N protein (*18*). When testing with this assay, anti-N positivity remains high over a substantial follow-up period; >94% of persons retain a positive value 10 months after infection (*19*,*20*). Thus, we assumed anti-N positivity to be evidence of any prior infection, not just recent infections.

Among specimens with anti-N, we selected a subset for further testing with a quantitative assay that determines the spike protein receptor-binding domain IgG (anti-RBD) level. That subset was selected because samples were received until either a target number for each patient age category and state of residence was reached or the data collection period concluded. Among specimens tested for quantitative anti-RBD titers, we tested a random subset of collected specimens for neutralizing antibodies within each age category. We determined subset size according to available resources.

### Quantitative Anti-RBD Assay

We used the electrochemiluminescent Cov2Quant IgG assay (LabCorp, https://www.labcorp.com) to quantify anti-RBD IgG in serum samples (*21*). We calculated the anti-RBD concentration of each serum sample by using a dilution series of affinity-purified human IgG standard and calibrated results with the World Health Organization international standard (international reference standard conversion factor for wild-type spike protein D614G mutation 50% infectious dose is 20/136) to convert results into binding antibody units (BAU) per milliliter. The threshold for anti-RBD positivity is 17.8 BAU/mL (sensitivity, 99.4% [95% CI 96.6%–99.9%]; specificity, 98.4% [95% CI 98.4%–99.6%]). For samples that returned a value <1.0, the lower quantitation limit of the assay, we used 1.0 as the value for quantitative results to enable statistic calculations on the logarithmic scale. Full methods are discussed elsewhere (*11*,*21*).

### Neutralizing Antibody Assay

We used the PhenoSense SARS-CoV-2 Neutralizing Antibody Assay (Mongram Biosciences, https://monogrambio.labcorp.com) (*22*,*23*) to measure neutralizing antibodies against the wild-type SARS-CoV-2 spike protein (GenBank accession no. MN908947.3). We recorded the 50% neutralization titer (NT_50_) as the reciprocal of the serum dilution conferring 50% inhibition of pseudovirus infection, with an upper limit of detection of 787,320 IU/mL. Full methods are discussed elsewhere (*10*,*23*).

### Statistical Analysis

We recorded counts and percents across covariates for anti-N seropositive samples, those with binding antibody titer results, and those with both binding and neutralizing antibody titer results. We compared distribution of age, sex, and metro status (i.e., living in a metropolitan statistical area or nonmetro area) among patient specimens with US Census Bureau estimates of the total US population in 2021 (*24*). We calculated Pearson correlation coefficients between log_10_-transformed quantitative binding and neutralizing antibody assay results stratified by age: children 0–17 years of age and adults >18 years of age. Then, we stratified binding and neutralization titers by sex assigned at birth (male or female) and age group (<18, 18–49, 50–64, or >65 years). We used a *t*-test on log_10_-transformed data to statistically compare mean titer by sex and a simple linear regression to examine whether slope, indicated by β, was nonzero across age groups. We conducted further analyses to compare binding and neutralizing titers by age group and 4 epochs of the pandemic, delineated by which SARS-CoV-2 variant was responsible for >50% of US cases: wild-type during January 1–March 27, 2021; Alpha during March 28–June 25, 2021; Delta during June 26–December 17, 2021; and Omicron BA 1.1 during December 8, 2021–February 28, 2022 (*25*). We performed 4 simple linear regressions for each assay result, 1 for each age group, to statistically described that trend. We then compared trends in binding antibody titer, neutralizing antibody titer, and national vaccination rates as recorded by CDC (*26*), for each age group over time. 

We performed all statistical analyses in R version 4.1.2 (The R Project for Statistical Computing, https://www.r-project.org). We considered p<0.05 statistically significant and did not correct for multiple comparisons. 

### Ethics Statement

This activity was reviewed by CDC and conducted consistent with applicable federal law and CDC policy (45 C.F.R. part 46, 21 C.F.R. part 56; 42 U.S.C. Sect. 241(d); 5 U.S.C. Sect. 552a; 44 U.S.C. Sect. 3501 et seq.). CDC determined that this project was public health surveillance and not research. Thus, approval by an institutional review board was waived. Informed consent was also waived because all data were deidentified.

## Results

**Figure 1:**
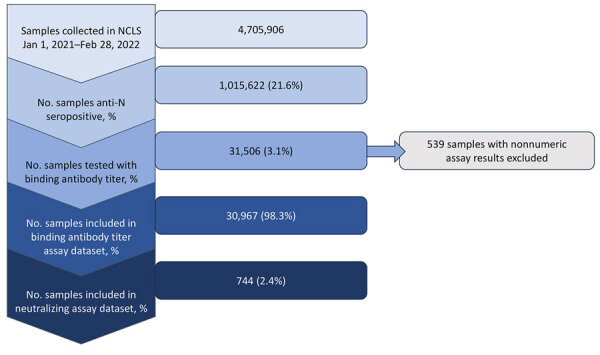
Flowchart of sample selection in study of quantitative SARS-CoV-2 spike receptor-binding domain and neutralizing antibody titers in previously infected persons, United States, January 2021–February 2022. Nonnumeric results are quantitative receptor-binding domain assays with invalid results due to insufficient serum volume, lost sample, or poor reproducibility. NCLS, Nationwide Commercial Laboratory Seroprevalence Survey.

During January 1, 2021–February 28, 2022, the NCLS study collected 4,705,906 serum specimens, of which 1,015,622 (21.6%) were anti-N seropositive (Figure 1). Of those seropositive samples, we tested 31,506 (3.2%) for binding antibody titer, of which 30,967 (98.3%) had valid numeric anti-RBD assay results. We included data from samples with valid assay results in our binding antibody titer analysis. Of the specimens included in the anti-RBD dataset, 744 (2.4%) had neutralizing titer results from the same specimen.

When we compared anti-N–positive samples in NCLS, samples with binding antibody titer results, and samples with neutralizing antibody titer results, we found that all 3 groups originated from populations who had similar distributions by sex and residence in a metropolitan area (Table). Percentages in each age group varied by dataset. Among anti-N–positive specimens, the lowest percentage (18%) was among persons >65 years of age. Because of the sampling design, nearly equal percentages of specimens were available by age group among those with binding antibody titers (age range <1–99 years). Among specimens with neutralizing antibody titers, the neutralization assay dataset, most samples were from persons in the youngest (<18 years) or oldest (>65) age groups (age range <1–93 years).

**Table:** Characteristics of subjects in a study of quantitative SARS-CoV-2 spike receptor-binding domain and neutralizing antibody titers in previously infected persons, United States, January 2021–February 2022*

Characteristics	% Total US population, 2021	Anti-N seropositive,† n = 1,015,622	Binding antibody titer available, n = 30,967	Neutralizing and binding antibody titers available, n = 744
Sex				
M	49.6	4.2 (41.3)	13,146 (42.5)	317 (42.6)
F	50.3	5.9 (58.1)	17,821 (57.5)	427 (57.4)
Age group, y				
<18	21.9	2.4 (23.6)	8,089 (26.1)	231 (31.0)
18–49	42.0	3.5 (34.5)	8,127 (26.2)	133 (17.8)
50–64	19.1	2.4 (23.6)	7,224 (23.3)	153 (20.5)
>65	16.9	1.8 (17.7)	7,527 (24.3)	227 (30.5)
Residence				
Nonmetro	13.8	1.7 (16.7)	5,767 (18.6)	136 (18.3)
Metro	86.2	8.4 (82.7)	25,200 (81.4)	608 (81.7)
Variant epoch				
Wild-type		2.5 (24.6)	9,049 (29.2)	202 (27.2)
Alpha		3.1 (30.5)	6,868 (22.2)	75 (10.1)
Delta		3.7 (36.4)	10,808 (34.9)	361 (48.5)
Omicron		0.9 (8.9)	4,242 (13.7)	106 (14.2)

*Values are no. (%) except as indicated. Population data are from the US Census Bureau (https://www.census.gov). Anti-N, nucleocapsid antibody. †Values are no. × 10^5^ (%).

**Figure 2:**
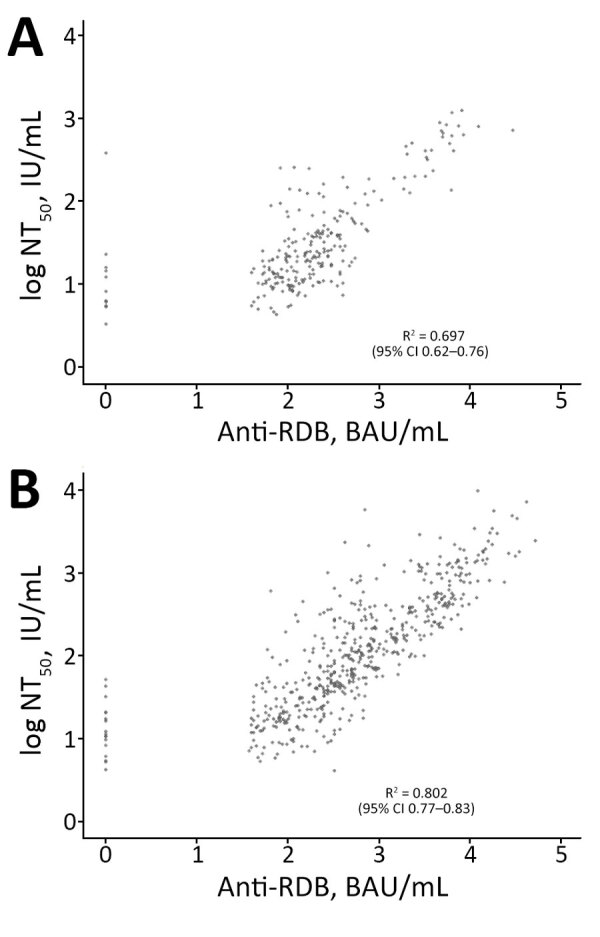
Pearson correlations in study of quantitative SARS-CoV-2 spike RBD and neutralizing antibody titers in previously infected persons, United States, January 2021–February 2022. A) Samples from children; B) samples from adults. Correlations are between the log_10_-transformed binding (anti-RBD IgG) and neutralizing antibody titers (NT_50_) for 744 specimens with available results in the Nationwide Commercial Laboratory Seroprevalence Survey database. Neutralizing antibody titers are against the ancestral spike protein. BAU, binding antibody units; NT_50_, 50% neutralization titer; RBD, receptor-binding domain.

The percentage of samples gathered during variant epochs also varied by dataset. The smallest percentage of specimens with anti-N (9%) and specimens with binding antibody titers (14%) were collected during the Omicron epoch but had representation of >20% from each earlier epoch. Specimens with neutralizing antibody titers were predominantly from the wild-type and Delta variant epochs and a lower percentage from the Alpha (10%) and Omicron (14%) epochs. Specimens collected from children and adults across all 4 variant epochs showed positive correlations between the log_10_-transformed binding and neutralizing antibody titers (children R^2^ = 0.70 [95% CI 0.62–0.76]; adult R^2^ = 0.80 [95% CI 0.77–0.83]) (Figure 2).

**Figure 3:**
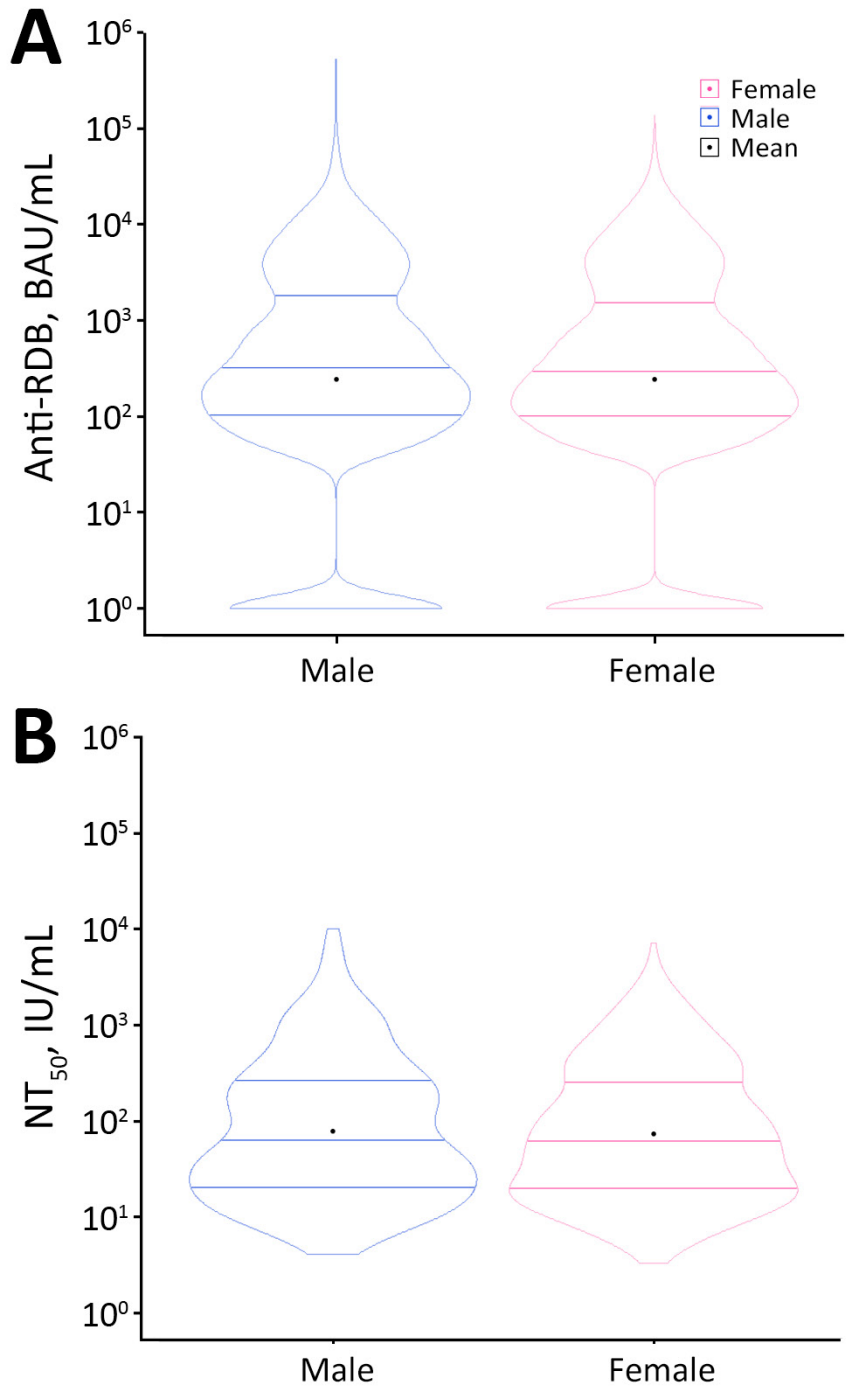
Violin plots of anti-RBD IgG and NT_50_ titer results by sex in study of quantitative SARS-CoV-2 spike RBD and neutralizing antibody titers in previously infected persons, United States, January 2021–February 2022. A) Anti-RBD, n = 30,967; B) NT_50_, n = 744. Anti-RBD measured by Cov2Quant IgG (LabCorp, https://www.labcorp.com) and NT_50_ measured by PhenoSense SARS-CoV-2 Neutralizing Antibody Assay (Monogram Biosciences, https://monogrambio.labcorp.com). Horizontal lines in plots indicate first quantile, median, and third quantile; black dots indicates means. BAU, binding antibody units; NT_50_, 50% neutralization titer; RBD, receptor-binding domain.

**Figure 4:**
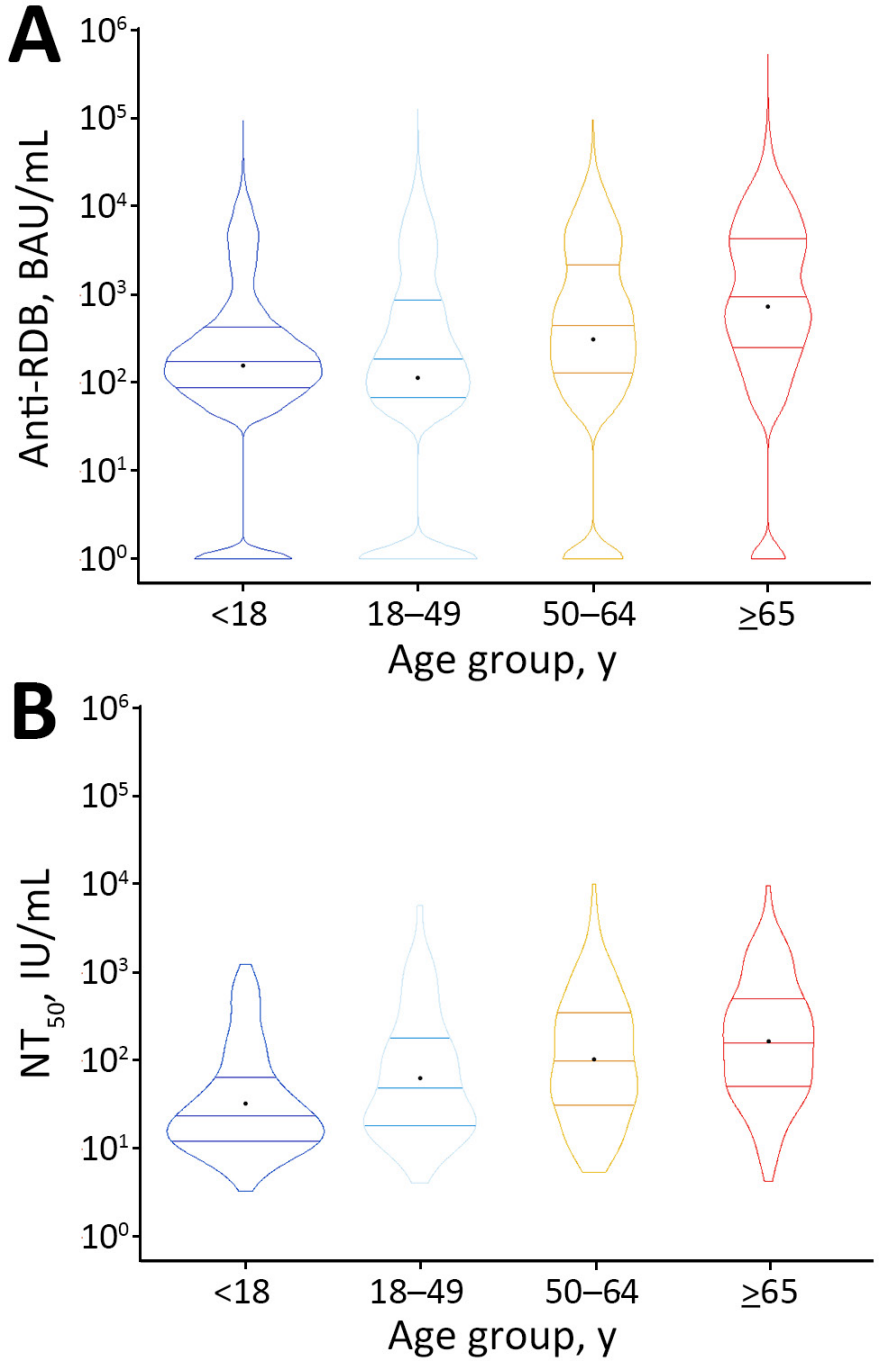
Violin plots of anti-RBD IgG and NT_50_ titer results by age group in a study of quantitative SARS-CoV-2 spike RBD and neutralizing antibody titers in previously infected persons, United States, January 2021–February 2022. A) Anti-RBD, n = 30,967; B) NT_50_, n = 744. Anti-RBD measured by Cov2Quant IgG (LabCorp, https://www.labcorp.com) and NT_50_ measured by PhenoSense SARS-CoV-2 Neutralizing Antibody Assay (Monogram Biosciences, https://monogrambio.labcorp.com). Horizontal lines in plots indicate first quantile, median, and third quantile; black dots indicates means. BAU, binding antibody units; NT_50_, 50% neutralization titer; RBD, receptor-binding domain.

We noted no clear difference in distribution in either binding or neutralizing titers between sexes (binding titers mean difference = 0.002 [95% CI −0.02 to 0.03]; neutralizing titers mean difference = 0.03 [95% CI −0.08 to 0.13]) (Figure 3). In contrast, mean titers had a notable positive trend across age groups for both titers (binding titers β = 0.24 [95% CI 0.23–0.25]; neutralizing titers β = 0.23 [95% CI 0.20–0.27]) (Figure 4).

**Figure 5:**
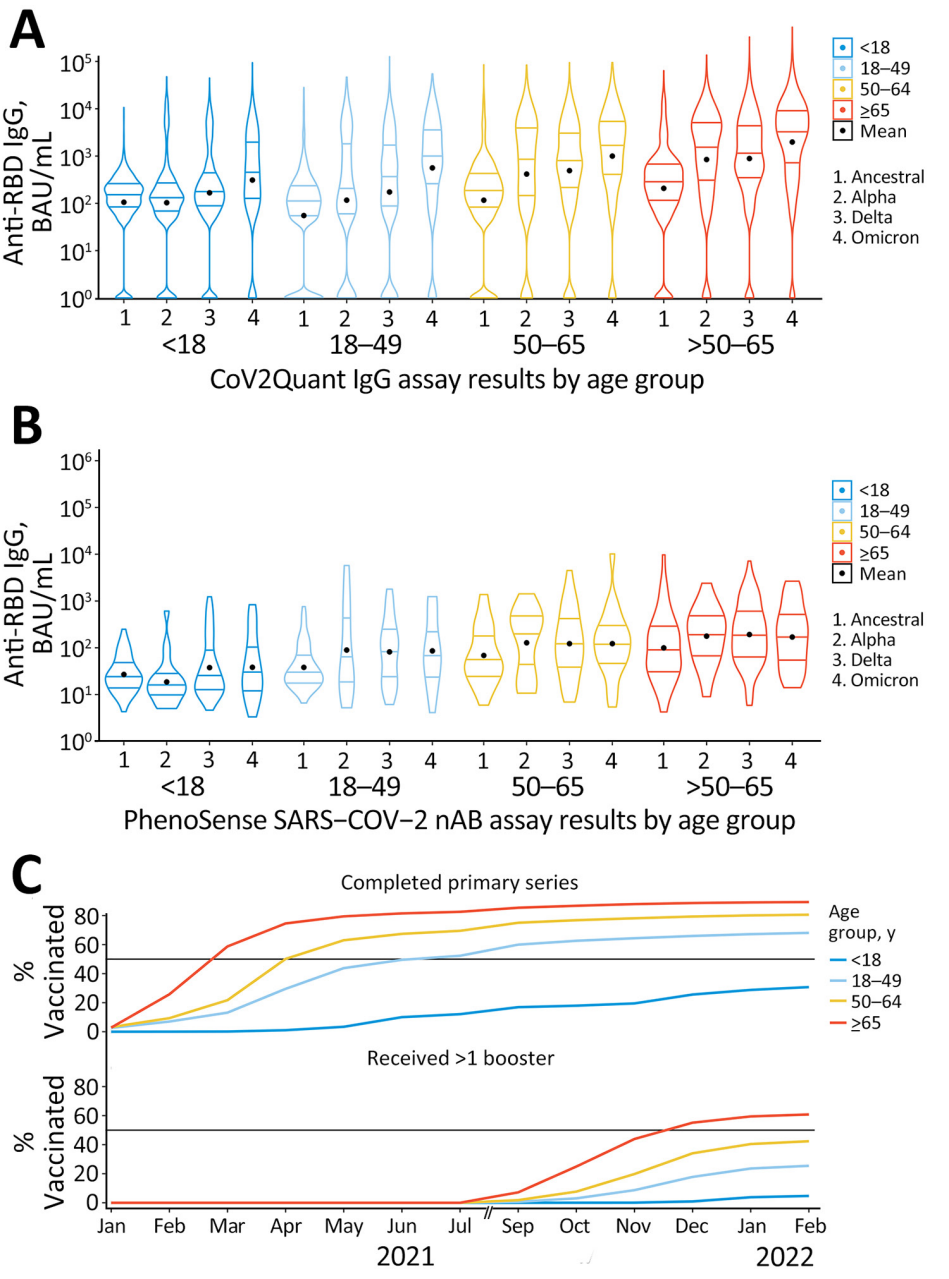
Violin plots of anti-RBD IgG and NT_50_ titer results by age group in study of quantitative SARS-CoV-2 spike RBD and neutralizing antibody titers in previously infected persons, United States, January 2021–February 2022. A) Anti-RBD, n = 30,967; B) NT_50_, n = 744; C) vaccination coverage per SARS-CoV-2 epoch. Anti-RBD measured by Cov2Quant IgG (LabCorp, https://www.labcorp.com) and NT_50_ measured by PhenoSense SARS-CoV-2 Neutralizing Antibody Assay (Monogram Biosciences, https://monogrambio.labcorp.com). Horizontal lines in plots indicate first quantile, median, and third quantile; black dots indicates means. Persons >16 years of age were eligible for vaccination starting in December 2020. In May 2021, vaccination was approved for persons 12–15 years of age. In November 2021, vaccination was approved for persons 5–11 years of age. Note: August 2021 is omitted due to a gap in data collection. BAU, binding antibody units; NT_50_, 50% neutralization titer; RBD, receptor-binding domain.

Violin plots of binding antibody titers showed an increasing trend in mean titer over the 4 variant epochs for all age groups: <18 years, β = 0.15 (95% CI 0.13–0.17); 18–49 years, β = 0.29 (95% CI 0.27–0.32); 50–64 years, β = 0.30 (95% CI 0.28–0.33); and >65 years, β = 0.29 (95% CI 0.27–0.32) (Figure 5, panel A). In addition, maximum values increased over time. Among all specimens, 11.9% had anti-RBD titers below the limit of detection: 1,275 (14.1%) in the wild-type epoch, 986 (14.4%) in the Alpha epoch, 1,101 (10.2%) in the Delta epoch, and 318 (7.5%) in the Omicron epoch. Of note, the distribution for persons <18 years of age appeared bimodal during the Alpha and Delta epochs before returning to a less bimodal shape during the Omicron epoch.

Neutralizing antibody titers showed an increasing trend in mean titer over the 4 variant epochs for all age groups. Mean titers showed statistical significance when tested for linear increases over time for persons 18–49 years of age (β = 0.014 [95% CI 0.03–0.24]) and those >65 years of age (β = 0.09 [95% CI 0.00–0.18]) (Figure 5, panel B). We noted no consistent changes in distribution shapes over time. Compared with temporal trends in US vaccination coverage by age, we found that distributions in assay results shifted upward as each corresponding age group experienced an increase in vaccination coverage (Figure 5, panel C; Appendix Table).

## Discussion

This analysis demonstrates the contribution of SARS-CoV-2 binding and neutralizing antibody titers in epidemiologic studies among populations in which nearly all persons have detectible antibodies. Using the range of titers detected in a population with 100% seroprevalence, we were able to study correlations between those 2 measures and compare the means, ranges, and shape of their distributions by demographic group and over time. The ability of those measures to detect serologic heterogeneity within populations with high rates of seroprevalence is key to studying correlates of protection in previously infected or vaccinated persons (*12*).

We observed a highly positive correlation between SARS-CoV-2 binding and neutralizing antibody titers among a sample of persons with serologic evidence of previous infection. This correlation is consistent with previous studies showing that binding antibody titers could be an acceptable proxy for neutralizing antibody titers, which can be costly and difficult to obtain (*27*,*28*). Although neutralizing antibody titers have been determined to be a correlate of protection for COVID-19 vaccines (*12*), the assays are resource intensive and, thus, are unlikely to be used routinely in a clinical setting (*27*,*28*). Furthermore, that correlation establishes the relationship within our sample, enabling results for both binding and neutralizing titers to lend context to one another. However, interpreting antibody titers should be approached with caution because no threshold above which a person is completely protected from infection or severe disease has been established. Antibody titers have a dose-response effect, and higher titers lead to lower chance of infection or severe disease (*9*–*11*; J.A. Cohen et al., unpub. data). Of note, antibody levels cannot completely describe the risk for severe COVID-19. Despite higher vaccine rates and higher antibody levels, older adults remain at higher risk for severe COVID-19 than young adults (*29*). Moreover, the protection associated with an antibody titer differs by variant and by assay (*30*).

We observed increases in mean and range across age groups for both binding and neutralizing antibody titers, and persons >65 years of age had the largest means and ranges. Conversely, neither binding nor neutralizing antibody titers showed meaningful heterogeneity across sex. Those observations are consistent with previous research on antibody titers. One previous study observed an association between increased age and an increase in antibody titers, possibly due to increasing likelihood of vaccination (*6*). Although some studies have shown lower antibody titers among vaccinated older adults (*31*–*33*), those adults likely received a higher number of total vaccine doses. That increase in vaccination could overcome the negative correlation of older age and postvaccine antibody levels to produce the effect seen here. Other studies failed to find evidence of variations in antibody titer by sex (*6*,*7*).

Among this serial cross-sectional study of previously infected persons, we observed an increase in mean binding and neutralizing antibody titers in all age groups over the course of the COVID-19 pandemic in the United States. Increasing antibody levels in the population may be one of the reasons that, as COVID-19 community transmission continues to surge, the rate of hospitalizations and deaths has decreased over time. In addition, increases in titers corresponded with increases in nationwide vaccination rates within each age group. Thus, our observed increases over time and by age are consistent with a higher likelihood of being vaccinated over time and in older age groups (*34*,*35*). Similarly, reinfection could also be contributing to increases in mean titer, especially because likelihood of reinfection increases over time and with later variants (*36*).

In addition to showing differences in means, our analysis visualized distribution shapes over the 4 variant epochs. Upon detailed inspection, the distribution for persons <18 years of age appeared more bimodal during the Alpha and Delta epochs before returning to a more unimodal shape during the Omicron epoch. During the Alpha and Delta epochs, we observed a large mode of titers at lower levels along with a smaller but visible secondary mode at a higher antibody concentration (*37*). That observation is consistent with the staggered vaccine rollout, in which various age groups <18 years of age became eligible at various dates. In our data from the Alpha and Delta epochs, titers from older children who were eligible for vaccination contributed to the upper mode. Visualization of binding antibody titer distributions also highlighted another characteristic: the lower tails of each distribution remained over epochs. Given that binding antibody titers below the limit of detection of the assay remained, some persons might have failed to mount a robust anti-RBD response to their infection, possibly related to mild or asymptomatic infections (*38*). Alternatively, some persons might have mounted an initial anti-RBD response but experienced titer waning over time (*38*), possibly correlated with an absence of vaccination or reinfection (*36*,*39*). Anti-RBD has been observed to wane faster than anti-N (*40*).

The first limitation of our analysis that race, ethnicity, and vaccination history of persons contributing specimens were not available from commercial laboratory data, precluding analysis with those variables. Second, we did not use information on prior infections, such as date of symptom onset or confirmation, because that information was only available for a small subset of persons. Similarly, we cannot generalize our findings to those who have never been infected, a valuable consideration for our comparison to nationwide vaccination rates. Third, sex, age, and metro status distribution among patients who provided specimens varied slightly from distributions among the general US population; however, we noted no extreme deviations signaling serious generalizability concerns. Finally, our neutralizing assay used a pseudovirion constructed by using the wild-type spike protein, which does not correlate with protection as closely as would neutralizing assays using variant-specific correlates. However, evidence suggests that neutralizing assays continue to correlate with protection against more recent variants (*41*,*42*)

In conclusion, binding and neutralizing antibody titers among persons with serologic evidence of prior SARS-CoV-2 infection have generally increased within age groups over variant epochs and in synchronization with increases in vaccination coverage. Those increases indicate a possible rise in protective immunity within age groups in previously infected populations over the study period, likely resulting from increases in vaccination coverage and reinfections. Our findings indicate that binding titers are acceptable in the absence of neutralizing titers in a clinical setting. In addition, our findings demonstrate the contribution of SARS-CoV-2 serologic surveys incorporating measures beyond seroprevalence for understanding immunity. Future serologic surveillance of infectious diseases would benefit from the incorporation of binding and neutralizing antibody titers. As the US population approaches 100% seroprevalence, those measures could help identify populations with less immunity to serve with vaccination campaigns.

Appendix Additional information on quantitative SARS-CoV-2 spike receptor-binding domain and neutralizing antibody titers in previously infected persons, United States, January 2021–February 2022.
