# Supplementary material for: Quantitative SARS-CoV-2 Spike Receptor-Binding Domain and Neutralizing Antibody Titers in Previously Infected Persons, United States, January 2021–February 2022
Source: Emerg Infect Dis. 2024 Nov;30(11):2352–61. doi: 10.3201/eid3011.240043 (PMC11521179; doi:10.3201/eid3011.240043)
Supplement: Appendix — Additional information on quantitative SARS-CoV-2 spike receptor-binding domain and neutralizing antibody titers in previously infected persons, United States, January 2021–February 2022. [file 24-0043-Techapp-s1.pdf]

Article DOI: https://doi.org/10.3201/eid3011.240043

EID cannot ensure accessibility for supplementary materials supplied by authors. Readers who have difficulty accessing supplementary content should contact the authors for assistance.

Quantitative SARS-CoV-2 Spike Receptor-binding Domain and Neutralizing Antibody Titers in Previously Infected Persons, United States, January 2021–February 2022

Appendix

Appendix Table. Means and medians for SARS-CoV-2 binding antibody titer (anti-RBD) and 50% neutralizing antibody titers (NT_{50}) by age and sex in the NCLS study, United States, January 2021–February 2022

Figure	Titer	Category		Mean	Median
3, panel A	Anti-RBD		Male	244	275
			Female	243	250
3, panel B	NT_{50}		Male	79	54
			Female	74	61
4, panel A	Anti-RBD		<18 y	154	165
			18–49 y	114	147
			50–64 y	308	375
			≥65 y	728	850
4, panel B	NT_{50}		<18 y	32	22
			18–49 y	63	47
			50–64 y	103	97
			≥65 y	164	160
5, panel A	Anti-RBD	<18 y	Ancestral	106	147
			Alpha	103	125
			Delta	167	167
			Omicron	310	400
		18–49 y	Ancestral	55	98
			Alpha	117	140
			Delta	173	275
			Omicron	562	875
		50–64 y	Ancestral	116	170
			Alpha	416	700
			Delta	491	725
			Omicron	998	1575
		≥65 y	Ancestral	208	275
			Alpha	844	1400
			Delta	885	1075
			Omicron	1977	3225
5, panel B	NT_{50}	<18 y	Ancestral	27	24
			Alpha	19	15
			Delta	38	23
			Omicron	38	22
		18–49 y	Ancestral	38	30
			Alpha	89	33
			Delta	82	102
			Omicron	86	67
		50–64 y	Ancestral	68	49
			Alpha	128	239
			Delta	122	117

Figure	Titer		Category	Mean	Median
			Omicron	123	125
		≥65 y	Ancestral	100	85
			Alpha	177	233
			Delta	192	177
			Omicron	170	175
